# Supplementary material for: Translational efficiency across healthy and tumor tissues is proliferation‐related
Source: Mol Syst Biol. 2020 Mar 9;16(3):e9275. doi: 10.15252/msb.20199275 (PMC7061310; doi:10.15252/msb.20199275)
Supplement: Supplementary file 1 — Expanded View Figures PDF [file MSB-16-e9275-s001.pdf]

Expanded View Figures

Figure EV1. Correlations between small RNA-seq and hydro-tRNAseq protocols, related to Fig 1.

A The Spearman correlations between all HEK293 tRNA quantifications, including four small RNA-seq datasets (Data ref: Flores *et al*, 2014b; Data ref: Mefferd *et al*, 2015b; Data ref: Torres *et al*, 2015b,c), four well-established tRNAseq datasets using Hydro-tRNAseq and DM-tRNAseq (Data ref: Zheng *et al*, 2015b; Data ref: Gogakos *et al*, 2017b; Data ref: Mattijssen *et al*, 2017b; Data ref: Benisty *et al*, 2019b), and one previously published quantification from small RNA-seq data by Zhang *et al* (2018). The scatter plots are square-root-normalized for better visualization.

B Principal component analysis (PCA) and linear discriminant analysis (LDA) of all absolute tRNA quantifications of HCT116, HELA, HEK293, and BJ fibroblasts, both from small RNA-seq and from Hydro-tRNAseq data. On the left, the first component of the PCA (method-related) explains 31.59% variance and the second component (cell-line-related) explains 30.83% variance. On the right, the first component of the LDA separates completely all four cell lines, regardless of the method.

 © 2020 The Authors

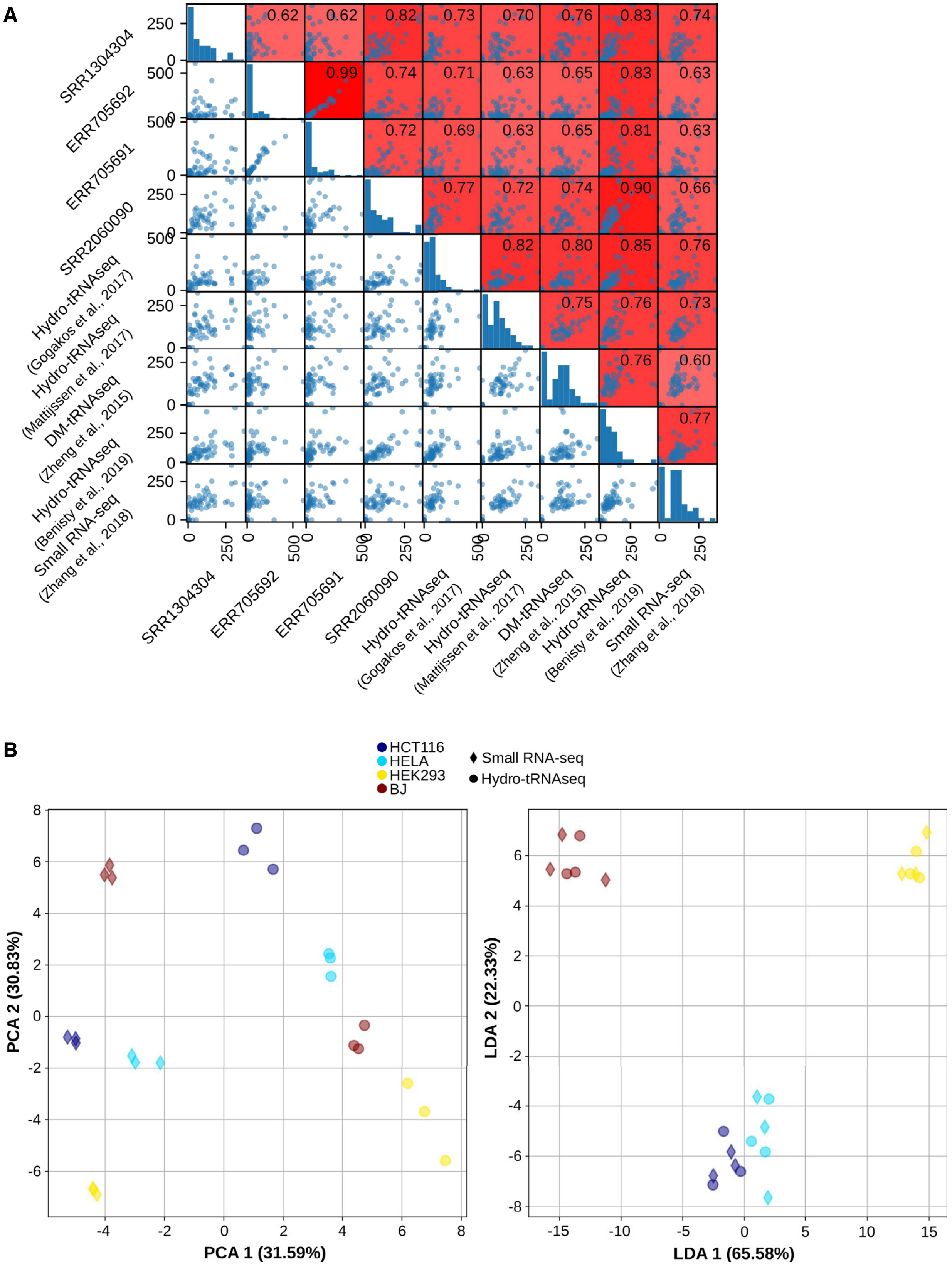
A
SRR1304304
ERR705692
ERR705691
SRR2060090
Hydro-tRNAseq (Gogakos et al., 2017)
Hydro-tRNAseq (Mattijssen et al., 2017)
DM-tRNAseq (Zheng et al., 2015)
Hydro-tRNAseq (Benisty et al., 2019)
Small RNA-seq (Zhang et al., 2018)
B
HCT116
HELA
HEK293
BJ
Small RNA-seq
Hydro-tRNAseq
PCA 1 (31.59%)
PCA 2 (30.83%)
LDA 1 (65.58%)
LDA 2 (22.33%)

Figure EV1.

Figure EV2. Comparison between raw sequencing data from small RNA-seq (left) and Hydro-tRNAseq (right), related to Fig 1.

A Length distribution of all reads mapping to tRNA genes.
B Fraction of the tRNA sequence that is covered by 1 or more reads, for each of the tRNA isoacceptors.
C Absolute number of reads mapping along the sequence of $tRNAPro^{AGG}$. The relative abundance between cell lines is not comparable given their different sequencing depths.

© 2020 The Authors

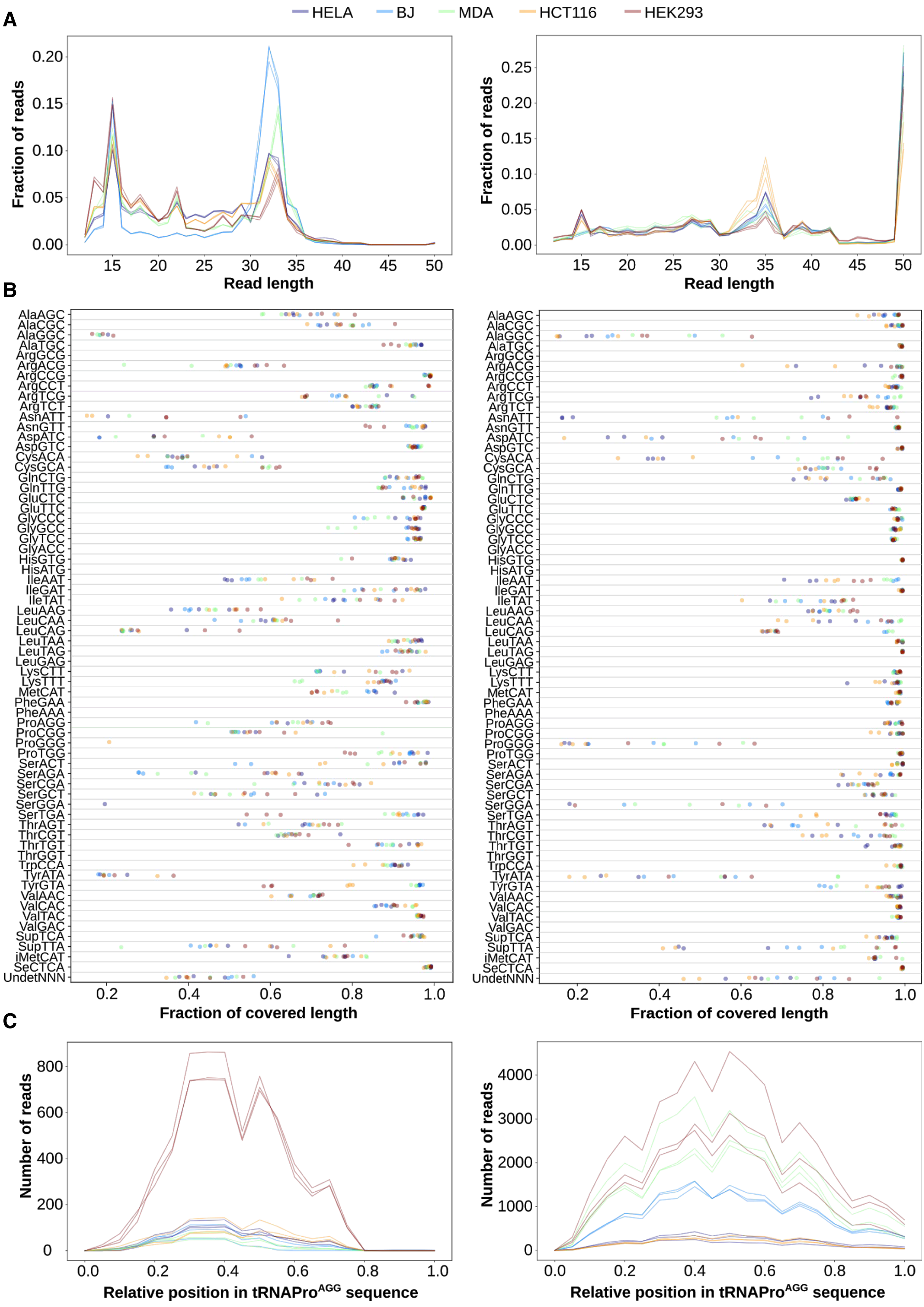
A
HELA
BJ
MDA
HCT116
HEK293
Fraction of reads
Read length
B
Fraction of covered length
C
Number of reads
Relative position in tRNAPro^{AGG} sequence

Figure EV2.

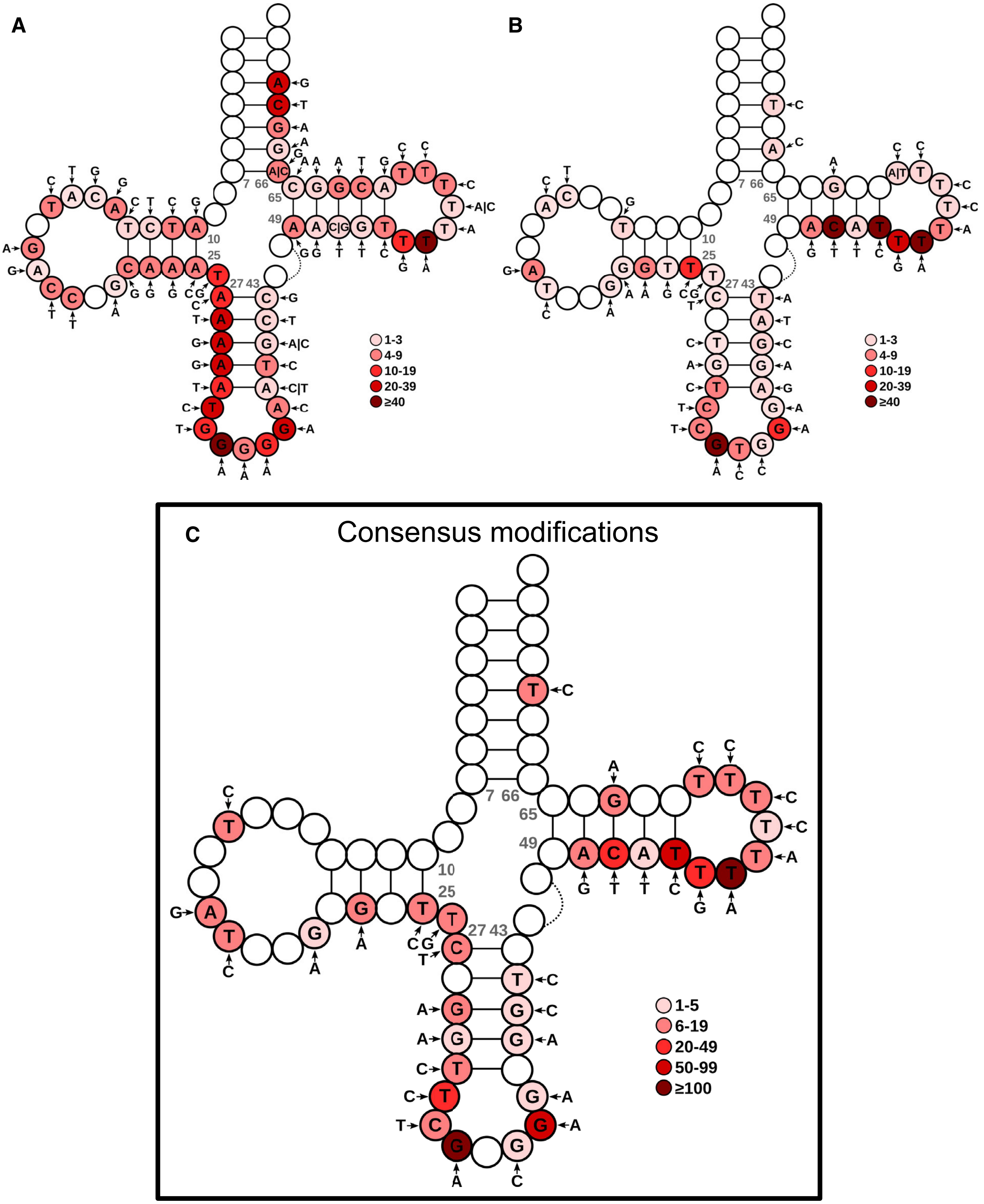

Figure EV3. Nucleotide modifications of HEK293 on the consensus tRNA structure, related to Fig 1.

A, B Over the consensus mature tRNA structure of tRNAdb (Jühling *et al*, 2009), the most frequent detected variants are depicted for both Hydro-tRNAseq (A) and small RNA-seq (B). The color code represents the number of tRNAs showing the modification at each specific position of the tRNA model.

C Consensus modifications detected by both methods. The color intensity refers to the sum of mismatch calls of the two methods altogether. Refer to Table EV3 for a detailed list of modifications for all cell lines.

 © 2020 The Authors

Figure EV4. Supply-to-Demand Adaptation across TCGA samples, related to Fig 3.

A Scatter plots against the proteome of the breast cancer sample TCGA-A2-A0T2-01A. Three metrics of translational efficiency (the classical tAI, a relative tAI with normalized weights within each amino acid family, and the Supply-to-Demand Adaptation described in this article) are plotted against two proxies of translation (protein abundance and protein-to-mRNA ratio). Pearson's correlation is only determined for protein abundance, since the assumption of normality is not fulfilled by the ratios.

B Pearson's correlations of all samples for which proteomics data are available (BRCA, COAD and READ). Center values represent the median. Statistical differences are determined by sample-paired two-tailed Wilcoxon rank-sum test (n = 219).

C Medians of Supply-to-Demand Adaptation weights (SDAw) across all TCGA tissues. The dendrograms show a hierarchical clustering among tissues (right) and among SDAw (top). The tissue labels correspond to the average Ki67 expression. Refer to Table EV4 for full cancer type names and number of samples.

© 2020 The Authors

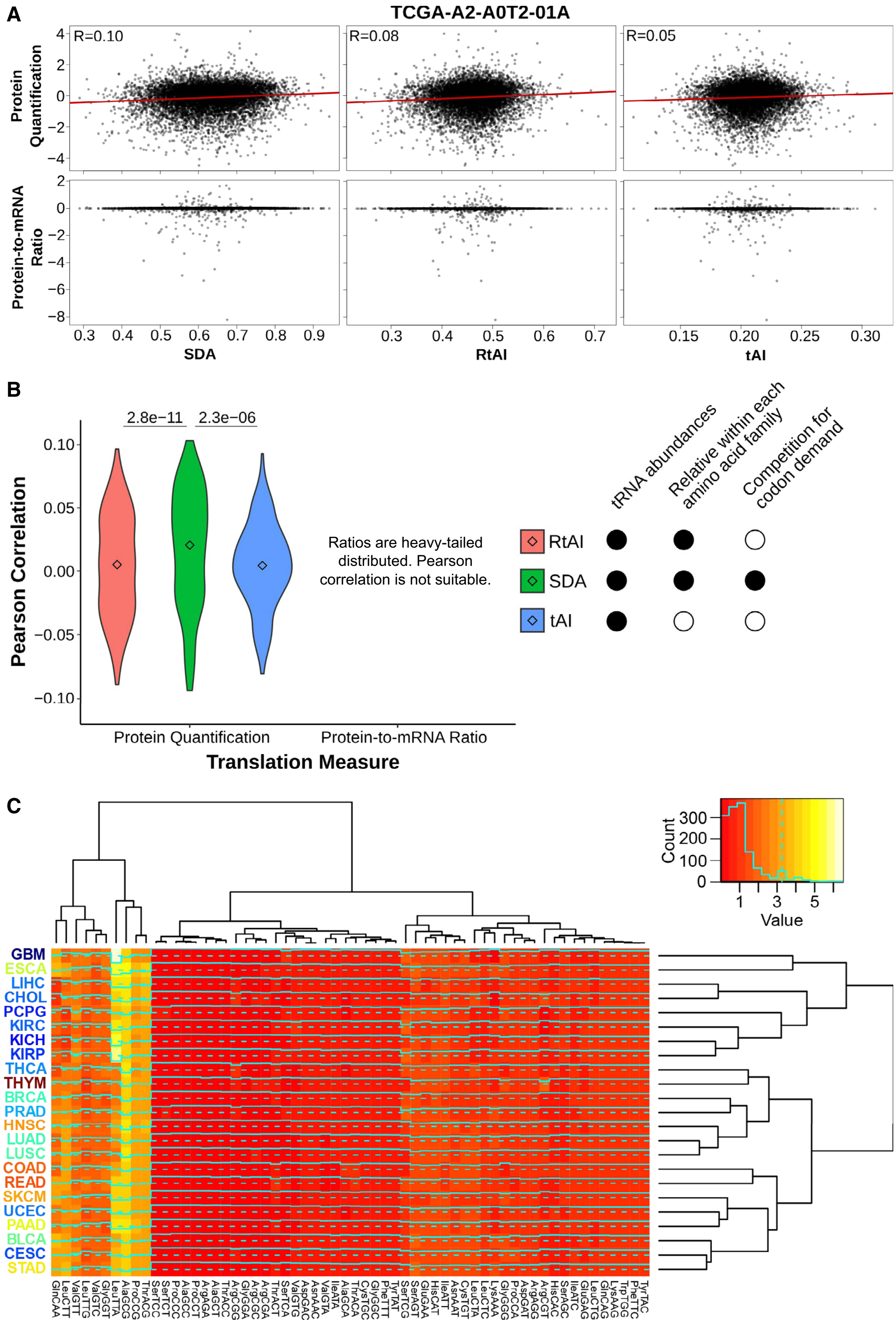

Figure EV4.

 © 2020 The Authors

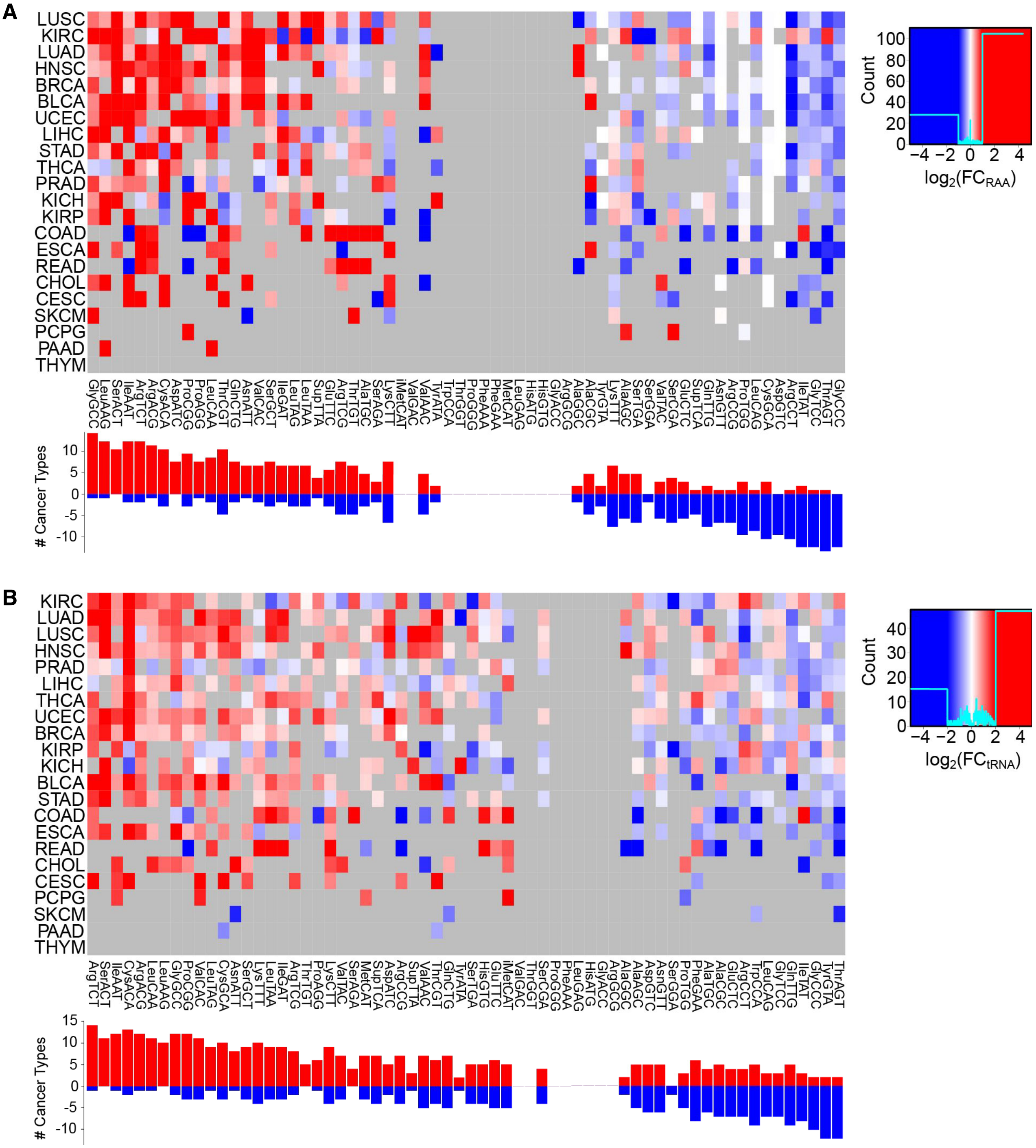

Figure EV5. Differential expression of tRNAs between healthy and tumor samples across 22 cancer types, related to Fig 4.

A tRNA abundance is measured as relative anticodon abundance, and the fold change is $\log_2(RAA_{Tumor}/RAA_{Healthy})$. Refer to Table EV4 for full cancer type names and number of samples.

B Absolute tRNA differential abundance by $\log_2(tRNA_{Tumor}/tRNA_{Healthy})$. Only significant differences are colored, which are determined using a two-tailed Wilcoxon rank-sum test and corrected for multiple testing by FDR.

Figure EV6. Differential methylation and copy number between healthy and tumor samples of tRNA genes, related to Fig 5.

A Differential average methylation (bisulfite sequencing) for the isoacceptors, averaged over all genes that have a certain anticodon. The difference is measured by Δ% Me = (%Me_{Tumor}-%$Me_{Healthy}$). Refer to Table EV4 for full cancer type names and number of samples.

B Differential average gene copy number for the isoacceptors, averaged over all genes that have a certain anticodon. The difference is measured by ΔCNA = (CNA_{Tumor}-$CNA_{Healthy}$). Only significant differences are colored, which are determined using a two-tailed Wilcoxon rank-sum test and corrected for multiple testing by FDR.

C Differential gene copy number between healthy and tumor samples of genes expressing proline tRNAs, as measured by ΔCNA. Only significant differences are colored, which are determined using a two-tailed Wilcoxon rank-sum test and corrected for multiple testing by FDR.

 © 2020 The Authors

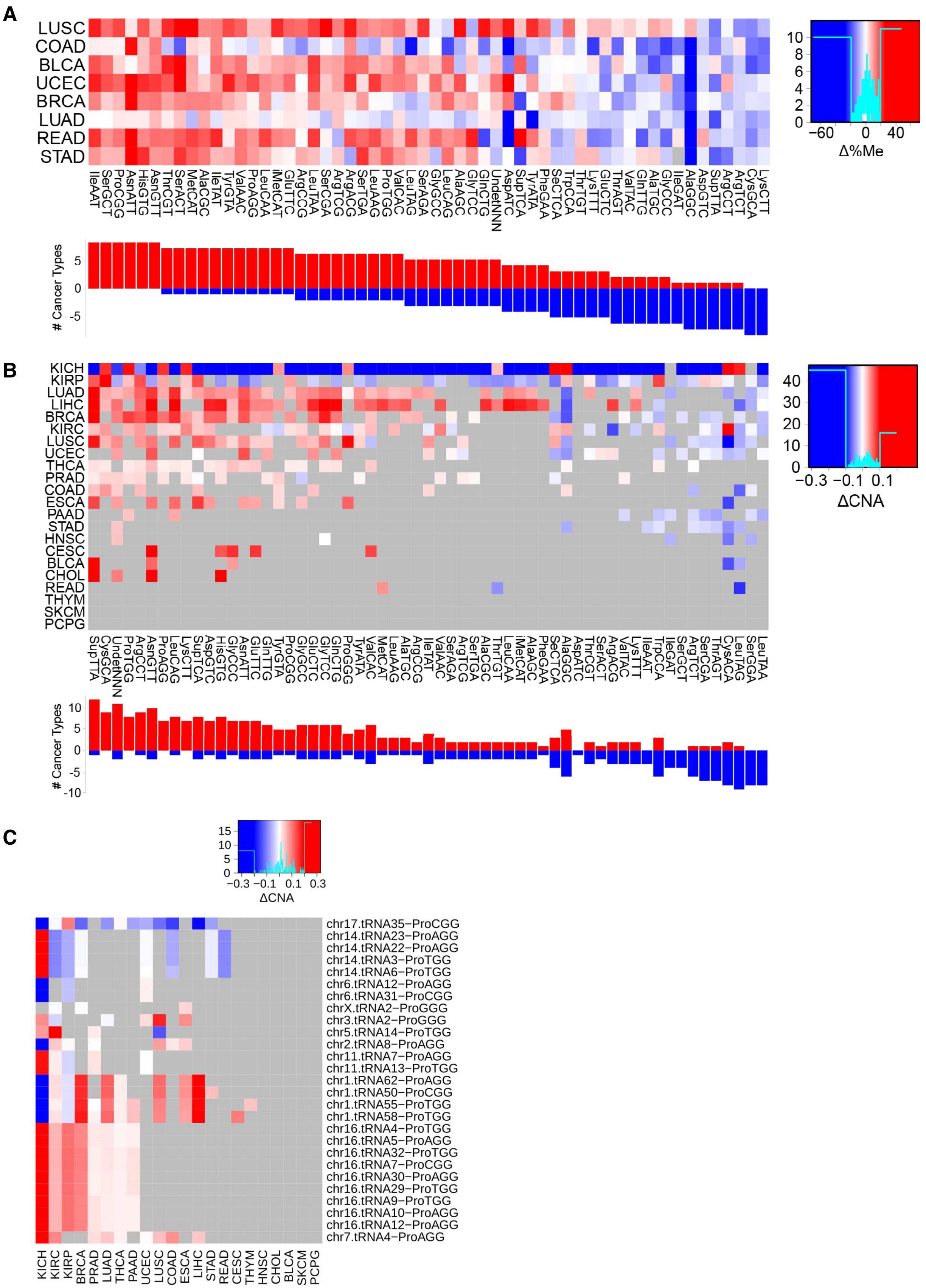
A
LUSC
COAD
BLCA
UCEC
BRCA
LUAD
READ
STAD
IleAAT SerGCT ProCGG AsnATT HisGTG AsnGTT ThrCGT SerACT MetCAT AlaCGC IleTAT TyrGTA ValAAC ProAGG LeuCAA iMetCAT GluTTC ArgCCG LeuTAA SerCGA ArgTCG ArgACG SerTGA LeuAAG ProTGG ValCAC LeuTAG SerAGA GlyGCC LeuCAG AlaAGC GlyTCC GlnCTG UndetNNN AspATC SupTCA TyrATA PheGAA SeCTCA TrpCCA ThrTGT LysTTT GluCTC ThrAGT ValTAC GlnTTG AlaTGC GlyCCC IleGAT AlaGGC AspGTC SupTTA ArgCCT ArgTCT CysGCA LysCTT
Δ%Me
Cancer Types
B
KICH
KIRP
LUAD
LIHC
BRCA
KIRC
LUSC
UCEC
THCA
PRAD
COAD
ESCA
PAAD
STAD
HNSC
CESC
BLCA
CHOL
READ
THYM
SKCM
PCPG
SupTTA CysGCA UndetNNN ProTGG ArgCCT AsnGTT ProAGG LeuCAG LysCTT SupTCA AspGTC HisGTG GlyCCC AsnATT GluTTC GlnTTG TyrGTA ProCGG GlyGCC GluCTC GlyTCC GlnCTG ProGGG TyrATA ValCAC MetCAT LeuAAG AlaTGC ArgCCG IleTAT ValAAC SerAGA ArgTCG SerTGA AlaCGC ThrTGT LeuCAA iMetCAT AlaAGC PheGAA SeCTCA AlaGGC AspATC ThrCGT SerACT ArgACG ValTAC LysTTT IleAAT TrpCCA IleGAT SerGCT ArgTCT SerCGA ThrAGT CysACA LeuTAG SerGGA LeuTAA
ΔCNA
Cancer Types
C
ΔCNA
chr17.tRNA35−ProCGG
chr14.tRNA23−ProAGG
chr14.tRNA22−ProAGG
chr14.tRNA3−ProTGG
chr14.tRNA6−ProTGG
chr6.tRNA12−ProAGG
chr6.tRNA31−ProCGG
chrX.tRNA2−ProGGG
chr3.tRNA2−ProGGG
chr5.tRNA14−ProTGG
chr2.tRNA8−ProAGG
chr11.tRNA7−ProAGG
chr11.tRNA13−ProTGG
chr1.tRNA62−ProAGG
chr1.tRNA50−ProCGG
chr1.tRNA55−ProTGG
chr1.tRNA58−ProTGG
chr16.tRNA4−ProTGG
chr16.tRNA5−ProAGG
chr16.tRNA32−ProTGG
chr16.tRNA7−ProCGG
chr16.tRNA30−ProAGG
chr16.tRNA29−ProTGG
chr16.tRNA9−ProTGG
chr16.tRNA10−ProAGG
chr16.tRNA12−ProAGG
chr7.tRNA4−ProAGG
KICH KIRC KIRP BRCA PRAD LUAD THCA PAAD UCEC LUSC COAD ESCA LIHC STAD READ CESC THYM HNSC CHOL BLCA SKCM PCPG

Figure EV6.
